# TGF-beta1 Gene Polymorphism in Association with Diabetic Retinopathy Susceptibility: A Systematic Review and Meta-Analysis

**DOI:** 10.1371/journal.pone.0094160

**Published:** 2014-04-07

**Authors:** Lei Liu, Jinghua Jiao, Yu Wang, Jingyang Wu, Desheng Huang, Weiping Teng, Lei Chen

**Affiliations:** 1 Department of Ophthalmology, The First Affiliated Hospital, China Medical University, Shenyang City, Liaoning Province, China; 2 Department of Anesthesiology, Fengtian Hospital, Shenyang Medical College, Shenyang City, Liaoning Province, China; 3 Department of Development and Planning office, China Medical University, Shenyang City, Liaoning Province, China; 4 Department of Epidemiology, School of Public Health, China Medical University, Shenyang City, Liaoning Province, China; 5 Department of Endocrinology and Metabolism, Institute of Endocrinology, Liaoning Provincial Key Laboratory of Endocrine Diseases, The First Affiliated Hospital of China Medical University, Shenyang City, Liaoning Province, China; Cedars-Sinai Medical Center; UCLA School of Medicine, United States of America

## Abstract

**Background:**

Transforming growth factor-beta (TGF-β1) gene has been regarded as an important mechanism in angiogenesis, endothelial cell proliferation, adhesion,and the deposition of extracellular matrix. The TGF-β1 gene may be involved in the development of diabetic retinopathy (DR) through disrupting angiogenesis. However, studies investigating the relationship between −509C/T and +869T/C(L10P) polymorphisms and DR yielded contradictory and inconclusive outcomes. In order to realize these ambiguous findings, a meta-analysis was performed to assess the association between the TGF-β1 gene polymorphisms and susceptibility to DR.

**Methodology/Principal Findings:**

We conducted a search of all English reports on studies for the association between the TGF-β1 gene polymorphisms and susceptibility to DR using Medline, the Cochrane Library, EMbase, Web of Science, Google (scholar), and all Chinese reports were identified manually and on-line using CBMDisc, Chongqing VIP database, and CNKI database. The strict selection criteria and exclusion criteria were determined, and odds ratios (ORs) with 95% confidence intervals (CIs) were used to assess the strength of associations. The fixed or random effect model was selected based on the heterogeneity test among studies. Publication bias was estimated using Begg's funnel plots and Egger's regression test.

**Results:**

A total of three studies were included in the meta-analysis and all included studies analyzed patients with type 2 diabetes. For +869T/C(L10P) polymorphism, significant association was observed in an allele model (L versus P: OR = 1.34, 95%CI = 1.03–1.73) and the recessive model (LL versus LP+PP: OR = 1.70, 95%CI = 1.13–2.56). As regards −509C/T polymorphism, no obvious associations were found for all genetic models.

**Conclusions:**

This meta-analysis suggested that the +869T/C(L10P) polymorphism in TGFβ1 gene would be a potential protect factor for DR. However, the −509C/T polymorphism is not associated with DR.

## Introduction

It has been widely accepted that diabetic retinopathy (DR) is one of the foremost causes of blindness in the working age population [1], characterized by angiogenesis in retina. However, the etiology of DR remains unknown and disease-modifying treatments are limited. In addition, since the involvement of cytokines in DR is hypothesized, there were many candidate genes approach in designing a case-control association study of single nucleotide polymorphisms (SNPs) including transforming growth factor-beta (TGF-β1) [2].

TGF-β1 has an important role in angiogenesis, endothelial cell proliferation, adhesion and the deposition of extracellular matrix [3], [4]. The TGF-β1 gene may be involved in the development of diabetic retinopathy (DR) through disrupting angiogenesis and blood retina barrier breakdown [5]. The highly polymorphic human TGF-β1 gene is located on chromosome 19q 13.1–13.3 [6]. There were some known TGF-β1 gene polymorphisms such as −988C/A, −800G/A, −509C/T and +869T/C(L10P).

Thus far, previous studies concerning association between −509C/T polymorphism and risk of DR are limited and rather conflicting within type 2 diabetes [7], [8], [9]. The second TGF gene polymorphic exchange +869T/C(L10P) is not commonly analyzed as −509C/T. Recently, few findings were frequently performed on the effect of +869T/C(L10P) polymorphism on DR, but the results were limited [7], [8]. Considering that a single study may lack the power to provide a reliable conclusion, we performed a meta-analysis on these eligible studies, to investigate the precise relationship between the TGF gene polymorphism and susceptibility to DR, which could have a much greater possibility of reaching reasonably strong conclusions.

## Methods

### Selection of eligible studies

Two reviewers (Lei Liu and Jinghua Jiao) independently scrutinized studies on the associations between TGFβ1 gene polymorphisms and DR. We searched Medline (Jan. 1st, 1946 to Oct. 31th, 2013), Embase (Jan. 1st, 1950 to Oct. 31th, 2013), the Cochrane Library (up to 2013, issue 10), Chinese Biological Medicine (Jan. 1st, 1978 to Oct. 31th, 2013), China National Knowledge Infrastructure (Jan. 1st, 1979 to Oct. 31th, 2013), Wang Fang Data (Jan. 1st, 1982 to Oct. 31th, 2013) and Chongqing VIP database (Jan. 1st, 1982 to Oct. 31th, 2013) databases using the terms “transforming growth factor beta 1 or transforming growth factorβ1 or TGF beta 1 or TGFβ1”, “diabetes or diabetic complications” and “polymorphism, variant or mutation”. We used the PubMed option “Related Articles” for each study to retrieve additional potentially relevant articles. Reference lists were checked and researchers contacted for additional literatures. Authors of publications were contacted when results were unclear or when relevant data were not reported. The search was done without restriction on language, but we only included published articles written in English or Chinese.

### Selection criteria

Studies were selected if they met the following criteria: (1) association study in sporadic DR subjects; (2) there were available data for TGFβ1 gene mutations with risk of DR, using a case-control or cohort design; (3) the genotype distribution in the controls of all studies should be in agreement with Hardy-Weinberg equilibrium (HWE); and (4) in the case of multiple publications from the same study group, the most complete and recent results were used.

### Exclusion criteria

The exclusion criteria were defined as: 1) useless data reported, genotype number or frequency not included; 2) abstracts, reviews and animal studies; and 3) genotype distribution in the control population not consistent with HWE.

### Data extraction

After excluding the overlap studies and including the additional ones, two investigators independently extracted data from each study with a standard fashion and entered into a common database. When discrepancies were appeared, all investigators were recruited to assess the data. The following information was collected: First author, year of publication, country, ethnicity, characteristics, study design, sample sizes of patients and controls, genotype numbers, minor allele frequency (MAF) and numbers (MAN), *P* value for HWE.

The review and analysis were guided to conduct by the PRISMA statement for preferred reporting of meta-analysis [10].

### Statistical analysis

Odds ratio (ORs) with 95% confidence intervals (CIs) for genotypes and alleles were used to assess the strength of association between TGFβ1 gene polymorphisms and DR. The ORs were performed for the allele contrasts, additive genetic model, as well as recessive genetic model and dominant genetic model, respectively. Heterogeneity was examined with *I^2^* statistic interpreted as the proportion of total variation contributed by between-study variation. If there was a statistical difference in terms of heterogeneity, the random effects model would be used to estimate the pooled ORs [11], [12]. Otherwise, the pooled ORs were estimated using the fixed effects model [13]. Sensitivity analysis was carried out by deleting one single study each time to examine the influence of individual data set on the pooled ORs. The possible publication bias was estimated with funnel plots and Egger's test. An asymmetric plot suggests a possible publication bias and the P value of Egger's test less than 0.05 was considered representative of statistically significant publication bias [14]. All statistical tests were performed with Comprehensive Meta-Analysis software version 2.0 (Biostat, Englewood Cliffs, I.N.J., USA) and RevMan version 5.0 (Review Manager, Copenhagen: The Nordic Cochrane Centre, The Cochrane Collaboration, 2010). All *P* values were two sided and a *P* value of smaller than 0.05 for any test was considered to be statistically significant.

## Results

### Study inclusion and characteristics

**Figure 1:**
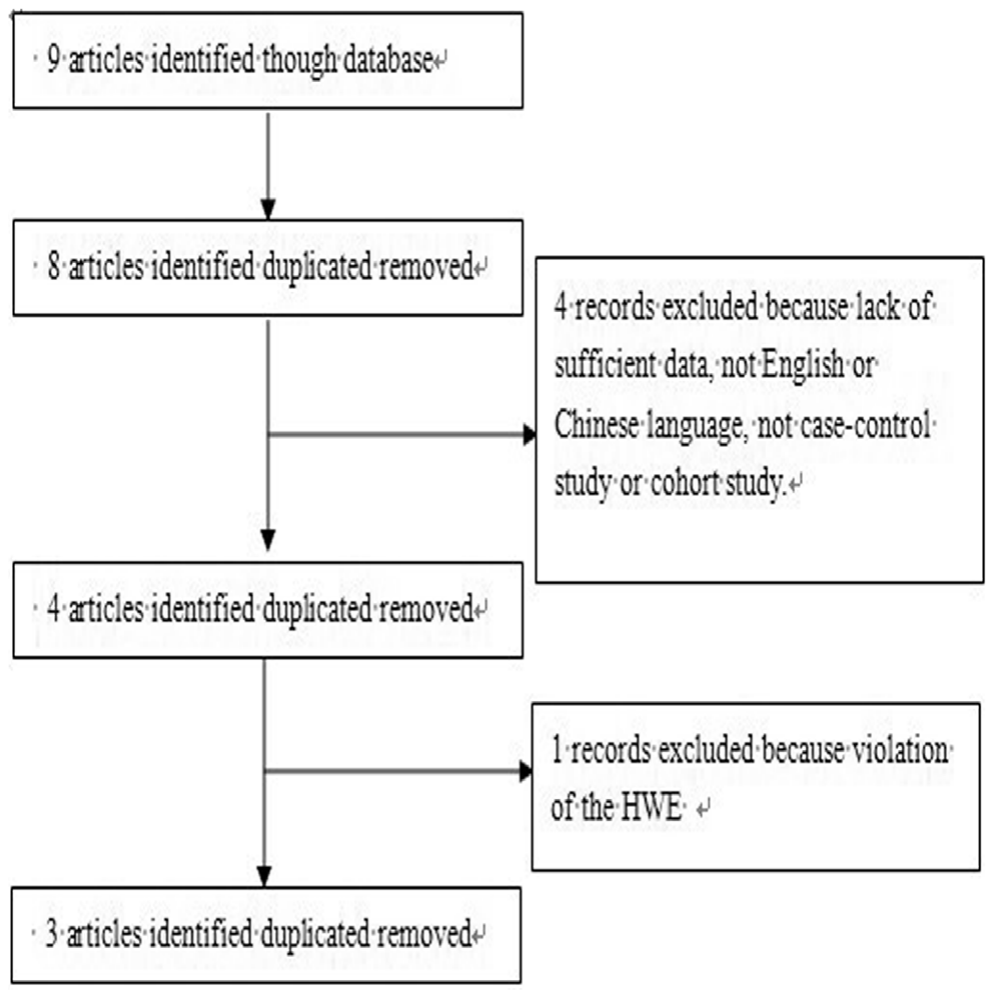
Flow chart demonstrating those studies that were processed for inclusion in the meta-analysis.

Finally, a total of three studies fulfilling the inclusion criteria were identified [7], [8], [9]. The results of the search are shown in Figure 1. However, patients in these three included studies were type 2 diabetes. In one of these studies, cases were recruited from 3 different countries, and thus each country in the literature was also considered separately for meta-analysis. Therefore, a total of five studies (three studies with 521 cases and 580 controls for −509C/T polymorphism and two studies with 268 cases and 340 controls for +869T/C(L10P) polymorphism) were included in the meta-analysis. The studies identified and their main characteristics are summarized in Table 1, Table 2 and Table 3. Genotype distribution of any polymorphism did not differ from Hardy-Weinberg equilibrium with in both groups (all were greater than 0.05).

**Table 1:** Characteristics of eligible studies included in the meta-analysis.

First author	Year	Country	Ethnicity	Study design	Criteria for DM	Criteria for DR	Polys	Cases			Controls		
								Subjects (women)	Age range (mean ± SD)	Grade of DR	Subjects (women)	Age range (mean ± SD)	Type
Beránek M [7]	2002	Czech	Caucasian	C-C	WHO	ETDRS	−988C/A, 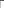 −800G/A, −509C/T, +869T/C(L10P)(L10P), +915G/C(R25P)	73(36)	48–79(62.9±8.1)	PDR	172(92)	24–90(62.6±12.1)	NIDDM
Buraczynska M [8]	2007	Poland	Caucasian	C-C	WHO	ETDRS	−509C/T, +869T/C(L10P), −396G/C	195(92)	N/A(58.6±9.4)	Any DR	168(87)	N/A(55.3±7.9)	Type 2 DM
Paine SK [9]	2012	India	Indian	C-C	WHO	ETDRS	−509C/T	253(120)	N/A(52±15.0)	PDR	240(112)	N/A(54±12.0)	Type 2 DM

Abbreviations: NIDDM: non-insulin-dependent diabetes mellitus. DM: diabetes mellitus. C-C: Case-control. PDR: proliferative diabetic retinopathy. DR: diabetic retinopathy. WHO: World Health Organization guidelines. ETDRS: the Early Treatment Diabetic Retinopathy Study. Polys: genotype combinations of polymorphisms. N/A: Not applicable.

**Table 2:** Distribution of −509C/T genotypes and allele frequencies among DR of cases and controls, and P-values of HWE in cases and controls.

First Auther	Number		Cases(N)			HWE	Controls(N)			HWE	C Allele(N)	
	case group	control group	CC	CT	TT	(p*value) for case group	CC	CT	TT	(p*value) for control group	case group	control group
Beránek M [7]	73	172	44	24	5	0.49	79	80	13	0.23	112	238
Buraczynska M [8]	195	168	79	85	31	0.31	40	44	16	0.51	243	124
Paine SK [9]	253	240	172	70	11	0.27	153	73	14	0.19	414	379

Abbreviations: DR: diabetic retinopathy; HWE: Hardy–Weinberg equilibrium.

**Table 3:** Distribution of +869T/C(L10P)(L10P) genotypes and allele frequencies among DR of cases and controls, and P-values of HWE in cases and controls.

First Auther	Number		Cases(N)			HWE	Controls(N)			HWE	C Allele(N)	
	case group	control group	LL	LP	PP	(p*value) for case group	LL	LP	PP	(p*value) for control group	case group	control group
Beránek M [7]	73	172	38	27	8	0.35	47	94	31	0.18	103	188
Buraczynska M [8]	195	168	43	86	66	0.14	22	44	34	0.28	172	88

Abbreviations: DR: diabetic retinopathy; HWE: Hardy–Weinberg equilibrium.

### Quantitative data synthesis

**Figure 2:**
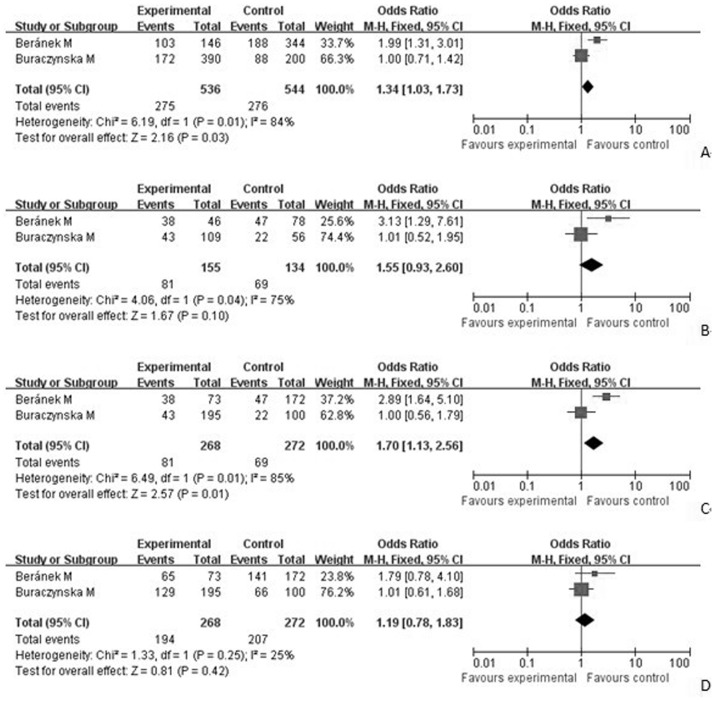
Forest plot of the association between DR and the TGFβ1 +869T/C(L10P) mutation (L vs P). Figure 2B. Forest plot of the association between DR and the TGFβ1 +869T/C(L10P) mutation (LL vs PP). Figure 2C. Forest plot of the association between DR and the TGFβ1 +869T/C(L10P) mutation (LL vs LP+PP). Figure 2D. Forest plot of the association between DR and the TGFβ1 +869T/C(L10P) mutation (LL+LP vs PP). Figure 3A. Forest plot of the association between DR and the TGFβ1 −509C/T mutation (C vs T). Figure 3B. Forest plot of the association between DR and the TGFβ1 −509C/T mutation (CC vs TT). Figure 3C. Forest plot of the association between DR and the TGFβ1 −509C/T mutation (CC vs CT+ TT). Figure 3D. Forest plot of the association between DR and the TGFβ1 −509C/T mutation (CC+CT vs TT).

As shown in Table 4, the results showed the evidence of an association between the decreased risk of DR and +869T/C(L10P) polymorphism in an allele model (L versus P: OR = 1.34, 95%CI = 1.03–1.73) and the recessive model (LL versus LP+PP: OR = 1.70, 95%CI = 1.13–2.56). There was no significant difference between-study heterogeneity (Figure 2).

**Table 4:** Summary ORs and 95%CI of the association between polymorphisms in the TGF β1 genes (−509C/T and +869T/C(L10P)(L10P)) and DR risk.

Gene	Allele contras		Additive model		Recessive model		Dominant model	
	OR(95%CI)	P	OR(95%CI)	P	OR(95%CI)	P	OR(95%CI)	P
+869T/C(L10P)	1.34(1.03–1/73)	0.03	1.55(0.93–2.60)	0.1	1.70(1.13–2.56)	0.01	1.19(0.78–1.83)	0.42
−509C/T	1.18(0.96–1.45)	0.11	1.23(0.76–1.99)	0.4	1.26(0.97–1.63)	0.09	1.13(0.72–1.79)	0.6

Abbreviations: DR: diabetic retinopathy; ORs: odds ratios; CI: confidence intervals.

**Figure 3:**
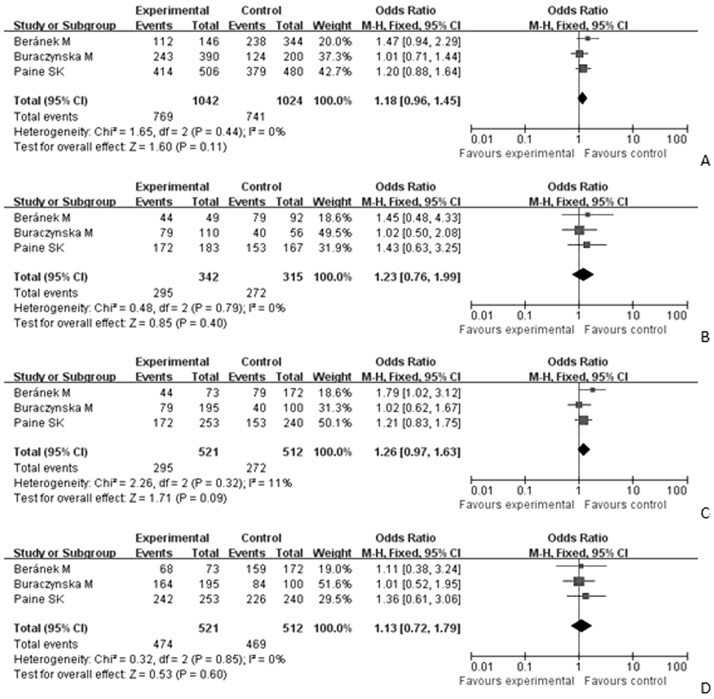
A. Forest plot of the association between DR and the TGFβ1 -509C/T mutation (C vs T). Figure 3B. Forest plot of the association between DR and the TGFβ1 -509C/T mutation (CC vs TT). Figure 3C. Forest plot of the association between DR and the TGFβ1 -509C/T mutation (CC vs CT+TT). Figure 3D. Forest plot of the association between DR and the TGFβ1 −509C/T mutation (CC+CT vs TT).

The association between −509C/T polymorphism and DR were also shown in Table 4. The results indicated no relationship of −509C/T polymorphism with DR risk (OR = 1.18, 95%CI = 0.96–1.45 for C versus T; OR = 1.23, 95%CI = 0.76–1.99 for CC versus TT; OR = 1.26, 95%CI = 0.97–1.63 for recessive model; OR = 1.13; 95%CI = 0.72–1.79 for dominant model) (Figure 3).

There was no significant publication bias according to Begg's and Egger's tests (Begg, p = 0.21; Egger, p = 0.71).

### Sensitivity analysis

In this study, we deleted every single study each time, in order to examining the influence of the individual data set to the pooled ORs. According to sensitivity analytical results, we found that there was no substantial modification of our estimates after exclusion of individual studies, indicating that the results were stable (data not shown).

## Discussion

There are three TGF-β isoforms, TGFβ1-3, which play important roles in regulating inflammation, cell growth and differentiation [15]. Previous research has revealed that TGFβ1 and TGFβ2 were profibrotic, but TGFβ3 might be antifibrotic [16]. Because there is little information on TGFβ2 and TGFβ3 with DR, a pertinent analysis of isoforms other than TGFβ1 in this meta-analysis has not been done.

Previous research suggested that +869T/C(L10P) gene polymorphism is associated with diabetic nephropathy in Chinese. The role of TGFβ1 in pathogenesis of diabetic nephropathy is pathologic tissue fibrosis leading to organ failure [17]. In addition, TGFβ1 is believed to have an important role in the pathogenesis of fibrotic diseases in the eye including proliferative vitreoretinopathy [18]. The relationship between TGFβ1 and the DR appears to be generation of exacerbation of angiogenesis and inhibiting the endothelial barrier function in the eye [19]. Therefore, the local activation of TGF-β1 expression may play an important role in the development of the phase of DR.

Except for evaluating the expression of TGFβ1 in vivo or in vitro [20], [21], there were few studies in DR patients that susceptibility to angiogenesis may be correlated with the presence of particular alleles at the TGF-β1 locus. However, the results are inconsistent and inconclusive due to limited sample size and different study populations. In previous study by Beránek M et al. [7], there is a marginally significant contrast by the −509C/T variant. But in the study by Paine SK et al. [9], −509C/T was no significantly associated with DR.

To the best of our knowledge, this is the first meta-analysis to explore TGFβ1 polymorphism in development and progression of DR. To achieve a more reliable and comprehensive conclusion on both variants, we used meta-analysis to assess the association between the TGFβ1 gene and DR risk on the basis of data from three studies. We summarized a significant association for +869T/C(L10P) polymorphism, especially in an allele model (L versus P: OR = 1.34, 95%CI = 1.03–1.73) and the recessive model (LL versus LP+PP: OR = 1.70, 95%CI = 1.13–2.56). Because the included studies were case-control studies, we did not perform a subgroup analysis by study design. However, the results indicated no relationship of −509C/T polymorphism with DR risk (OR = 1.18, 95%CI = 0.96–1.45 for C versus T; OR = 1.23, 95%CI = 0.76–1.99 for CC versus TT; OR = 1.26, 95%CI = 0.97–1.63 for recessive model; OR = 1.13; 95%CI = 0.72–1.79 for dominant model). The results from our study seem to indicate that the −509C/T polymorphism may be not associated with DR risk. Because heterogeneity was found among the studies, we employed random-effect model. Then a sensitivity analysis was carried out by removing one study for each time and re-running the model to determine the effect on the overall estimate. The estimates changed quite little, indicating that the results were stable. Owing to the limited number of included studies, we did not perform a publication bias test.

In addition, some other TGFβ1 genes including −988C/A, −800G/A, and +915G/C(R25P) were studied in the relationship with DR. The −988A allele was not represented in the study and no signicant differences between groups were found for the −800G/A. However, +915G/C(R25P) polymorphisms in the TGF-β1 gene could be regarded as a strong genetic risk factor for DR [7]. As only one study was reported for in these candidate genes, we could not use meta-analysis to analyze the relationship between these genes and DR.

In this meta-analysis, all included studies analyzed patients with type 2 diabetes. The etiology and mechanisms of type 1 and 2 diabetes are different, so there may be diversity in the association between TGFβ1 gene polymorphism and DR with type 1 or 2 diabetes. However, the relationship between TGFβ1 gene polymorphism and DR within type 1 diabetes is not demonstrated. More efforts are encouraged to explore this association.

This meta-analysis has pooled all the available results from the case-control studies, which has significantly increased the statistical power. However, the results of the present meta-analysis should also be interpreted within the context of its limitations. First, DR is a multi-factorial disease from complex interactions between environmental exposures and genes factors. In this meta-analysis, we had insufficient data to perform an evaluation of such interactions for the independent role of TGFβ1 polymorphisms in DR development. Second, the major limitation of our study is the relatively limited available studies included in the meta-analysis. Thus, investigations involving large size of different races are necessary for a more reliable evaluation on their associations. Third, our meta-analysis is based on unadjusted estimates because of a lack of original data. Forth, although every effort was made to ascertain all appropriate publications, it is likely that some were missed or displayed erroneously. In addition, we did not consider studies published in languages other than English/Chinese or data presented in abstracted form.

In spite of these limitations, for DR candidate genes, we believe that the positive locus identified in our systematic meta-analyses that warrant follow-up with high priority with a prospective trial.

In conclusion, this meta-analysis suggested that the +869T/C(L10P) polymorphisms in the TGF-β1 gene could be regarded as a strong genetic risk factor for DR. However, the −509T/C polymorphism is not associated with DR risk. At the same time, this result should be interpreted cautiously. To better understand the potential mechanism for DR in humans, in the future, large well-designed epidemiological studies in the susceptibility of DR evidence are needed to confirm this association. It also will be necessary to combine genetic factors and other environmental risk factors.

## Supporting Information

Checklist S1 PRISMA 2009 Checklist.
(DOC)
